# Artificial MicroRNA-Mediated Inhibition of Japanese Encephalitis Virus Replication in Neuronal Cells

**DOI:** 10.1089/nat.2018.0743

**Published:** 2018-11-30

**Authors:** Himani Sharma, Aarti Tripathi, Bharti Kumari, Sudhanshu Vrati, Arup Banerjee

**Affiliations:** ^1^ Vaccine and Infectious Disease Research Center (VIDRC), Translational Health Science and Technology Institute (THSTI), Faridabad, India.; ^2^ Regional Center for Biotechnology, NCR Biotech Science Cluster, Faridabad, India.

**Keywords:** JEV, artificial microRNA, replication, 3′UTR

## Abstract

Artificial microRNA (amiRNA)-mediated inhibition of viral replication has recently gained importance as a strategy for antiviral therapy. In this study, we evaluated the benefit of using the amiRNA vector against Japanese encephalitis virus (JEV). We designed three single amiRNA sequences against the consensus sequence of 3′ untranslated region (3′UTR) of JEV and tested their efficacy against cell culture-grown JEV Vellore strain (P20778) in neuronal cells. The binding ability of three amiRNAs on 3′UTR region was tested *in vitro* in HEK293T cells using a JEV 3′UTR tagged with luciferase reporter vector. Transient transfection of amiRNAs was nontoxic to cells as evident from the MTT assay and caused minimal induction in interferon-stimulated gene expression. Furthermore, our result suggested that transient expression of two amiRNAs (amiRNA #1 and amiRNA #2) significantly reduced intracellular viral RNA and nonstructural 1 (NS1) protein, as well as diminished infectious viral particle release up to 95% in the culture supernatant as evident from viral plaque reduction assay. Overall, our results indicated that RNA interference based on amiRNAs targeting viral conserved regions at 3′UTR was a useful approach for improvements of nucleic acid inhibitors against JEV.

## Introduction

RNA interference (RNAi) based on artificial microRNA (amiRNA)-mediated inhibition of the target gene has evidenced to be an essential tool for antiviral therapy. The amiRNAs are small RNA molecules that are expressed under the backbone of endogenous cellular miRNAs and act as more efficient inhibitors of cellular genes [1]. The amiRNAs express through RNA polymerase II. Like miRNAs, amiRNAs binds to the targeted mRNA in a sequence-specific manner leading to direct mRNA degradation or translational inhibition. Ideally, amiRNA-expressing vectors provide unique benefits in designing antiviral therapy as it is less toxic than regular shRNA vector [2–4]. Owing to the specificity and efficiency of gene silencing, it was increasingly investigated whether amiRNAs targeted to the viral genome can control viral replication in infected cells. So far, the amiRNA-mediated antiviral approach has found to be useful for many viruses, including adenoviruses, rabies virus, dengue virus, chikungunya, and porcine reproductive and respiratory virus. In all the cases, it has been shown to be an efficient inhibitor of virus replication with minimal or no cytotoxicity [5–8].

Japanese encephalitis virus (JEV) belongs to the Flaviviridae family causing viral encephalitis worldwide. It is estimated that 3 billion people living in 24 countries in the WHO Southeast Asia and Western Pacific regions are at risk of Japanese encephalitis (JE) [9]. In India, epidemics of JE reported from many parts of the country, and it is considered a major pediatric problem. Notably, in India, vaccination with an attenuated and genetically stable strain SA-14-14-2 started in 2006. However, the efficacy of the vaccine is reported to be less compared to other countries [10]. According to the National Vector-borne Disease Control Program (NVBDCP), JE accounted for 13.7% of total 63,854 acute encephalitis syndrome cases reported during 2010–2017 and was associated with a case fatality ratio of 17.3% [9]. Therefore, alternative antiviral strategies are required to control JEV.

The JEV genome is a positive-sense, single-stranded RNA of ∼11 kb. The genome consists of a single open reading frame flanked by 5′ and 3′ noncoding regions. Several attempts were made earlier to target the viral proteins to inhibit JEV replication using different methods with varying success [11–14].

In this study, we have targeted the viral 3′UTR region, which plays a critical role in the viral replication in the host cells. The 3′UTR has been shown to be necessary for viral replication and immune modulation. This region consists of elements that are essential for genome cyclization, resulting in the initiation of RNA synthesis [15]. In addition to the critical role 3′UTR plays on RNA synthesis, the stem-loop structure of 3′UTR is also responsible for the generation of subgenomic flavivirus RNA (sfRNA). This sfRNA helps the virus evade host immune response, thereby affecting viral pathogenesis. Therefore, to inhibit the viral replication, we designed amiRNAs against JEV 3′UTR and tested against cell culture-grown JEV strain. Our results suggested that targeting JEV 3′UTR by specific amiRNAs could be useful to reduce viral replication in the neuronal cells.

## Materials and Methods

### Cells

Porcine stable (PS) kidney cell line, mouse neuroblastoma cells (N2a), and human embryonic kidney-derived cell line (HEK293T) were procured from National Centre for Cell Science, Pune, India. PS cells were cultured in minimum essential medium media (HyClone; GE Healthcare Life Sciences, UT). The N2a and HEK293T cells were cultured and propagated in Dulbecco's modified Eagle's medium (HyClone; GE Healthcare Life Sciences). All the cells were cultured in complete media supplemented with 10% fetal bovine serum (HyClone), penicillin (100 U/mL), and streptomycin (100 g/mL).

### amiRNA design and plasmid construction

**FIG. 1.**
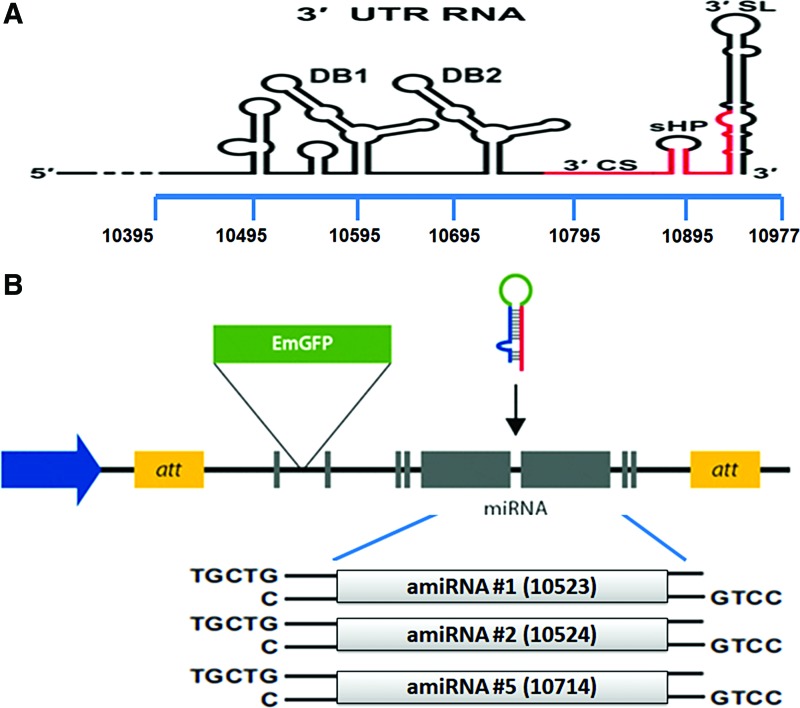
Cloning of amiRNA into pcDNA™6.2-GW/EmGFP-miR vector. **(A)** Schematic representations of the JEV 3*′*UTR structure. **(B)** Schematic illustrations of the three amiRNA designed against the target sites cloned into pcDNA6.2-GW/EmGFP-miR vector. amiRNA, artificial microRNA; JEV, Japanese encephalitis virus; UTR, untranslated region. Color images available online at www.liebertpub.com/nat

amiRNA sequences were designed using Block-iT RNAi Web Designer tool (Invitrogen, Inc., Carlsbad, CA). We aligned 3′UTR genomic sequences of the different genotypes of JEV strains [(1) Vellore P20778 (AF080251), (2) GP78 (AF075723), (3) SA14-14-2 (AF315119.1), (4) Nakayama strain (EF571853), (5) isolate JEV/SW/GZ/09/2004 China (KF297916), and (6) isolate JEV/eq/India/H225/2009 (JX131374)], and then designed amiRNAs targeting the highly conserved regions across the JEV 3′UTR by using Invitrogen online tool Block-iT RNAi Designer (http://rnaidesigner.invitrogen.com/rnaiexpress). We selected top three high scoring amiRNA sequences and the oligos were synthesized (Sigma) along with a control sequence, which was not specific against JEV 3′UTR sequence (Fig. 1A and Table 1). amiRNAs were cloned into pcDNA™6.2-GW/EmGFP-miR vector (Invitrogen, Inc.) according to the manufacturer's protocol (Fig. 1A). The amiRNA expressed was based on the natural structure of *Mus musculus* miR-155. An amiRNA targeting LacZ gene was also included as a negative control (NC) in this study. To avoid off-target effects, all of these amiRNA sequences were analyzed using NCBI Blastn against human and mouse transcript sequences.

**Table 1.** Oligonucleotides Designed for Artificial microRNA Construction

	*Position*	*Sequence (5′-3′)*
AmiRNA #1	10523_top	TGCTG*AGATTTGTTAACCCAGTCCTC*GTTTTGGCCACTGACTGACGAGGACTGTTAACAAATCT
	10523_bottom	CCTGAGATTTGTTAACAGTCCTCGTCAGTCAGTGGCCAAAACGAGGACTGGGTTAACAAATCTC
AmiRNA #2	10524_top	TGCTG*CAGATTTGTTAACCCAGTCCTG*TTTTGGCCACTGACTGACAGGACTGGTAACAAATCTG
	10524_bottom	CCTGCAGATTTGTTACCAGTCCTGTCAGTCAGTGGCCAAAACAGGACTGGGTTAACAAATCTGC
AmiRNA #5	10714_top	TGCTG*ATTGCATCCTAGACGAGGCTT*GTTTTGGCCACTGACTGACAAGCCTTGTAGGATGCAAT
	10714_bottom	CCTGATTGCATCCTACAAGGCTTGTCAGTCAGTGGCCAAAACAAGCCTTGTCTAGGATGCAATC
AmiRNA-lacZ	Top	TGCTGAAATCGCTGATTTGTGTAGTCGTTTTGGCCACTGACTGACGACTACACATCAGCGATTT
	Bottom	CCTGAAATCGCTGATGTGTAGTCGTCAGTCAGTGGCCAAAACGACTACACAAATCAGCGATTTC

### JEV infection and amiRNA transfection

N2a cells were plated on 6-well plates at a density of 1.5 × 10^5^ cells per well for 24 h before infection. The cells were infected with JEV [multiplicity of infection (MOI) of 5] as mentioned above. One microgram amiRNA of each clone was transfected into cells after 3 h of JEV infection using Lipofectamine 2000 reagent (Invitrogen) according to the manufacturer's protocol. Cells were allowed to grow up to 48 h. After 48 h postinfection (hpi), the culture supernatant was collected and used to determine infectious viral particle using plaque assay.

### Cell viability assay

N2a cells were plated on 96-well plates at a density of 1 × 10^4^ cells per well for 24 h. Cells were first infected and then transfected with the individual amiRNA as mentioned above. Cell Titer 96 Aqueous one solution reagent (20 μL; CellTiter 96^®^ Non-Radioactive Cell Proliferation Assay; Promega, Madison, WI) was added to each well 4 h before the duration of 48 hpi. Absorbance was measured at 570 nm using a 96-well multimode plate reader (Synergy 2Multi-Mode Reader; BioteK).

### Virus plaque assay

The JEV, Vellore strain (P20778), was propagated in PS cell and titrated by plaque assay [16]. Briefly, the PS cells were seeded in 12-well tissue culture plates at a density of 0.2 × 10^5^ cells per well. Cells were infected for 1 h with serially diluted cell culture-grown JEV (P20778) virus. Cells were rinsed twice with 1 × phosphate-buffered saline (PBS) to remove the excess virus. Then, the 2% low-melting agarose/growth media mixture (1:1) was added to each well. The plate was allowed to keep for 15 min at room temperature (RT) until the agarose overlay solidified. After growing for 3 days at 37°C with 5% CO_2_, cells were fixed with 4% formaldehyde at 37°C for 1 h. Cells were stained for 15 min at 37°C by adding 1.5% crystal violet solution. Cells were washed thrice with RO water, as above, and then plaques were counted to calculate the concentration of virus in plaque-forming units (PFU) per mL. [PFU = *N* × DF/*V* (*N*, number of plaques; DF, virus dilution factor; and *V*, the volume of the inoculum)].

### Construction of JEV 3′UTR luciferase reporter vector

We cloned double-stranded 3′UTR (P20778) containing miRNA target sequences into the *Xho*I-/*No*tI-digested psiCHECK-2 Dual-Luciferase Expression Vector (Promega). Sequencing confirmed the insert. HEK293T cells were cotransfected with 100 ng of the psiCHECK2 constructs and 1,000 ng of amiRNA plasmid and control plasmid, or different concentration of plasmid in each well of a 12-well plate. Luciferase readout was measured after 48 h of transfection.

### Cell-based indirect immunofluorescence assay

N2a cells were grown on the coverslip in a 24-well plate. After 24 h, cells were first infected with JEV (MOI = 5), and after 3 hpi, cells were transfected with amiRNAs. The culture medium was removed at 48 hpi. The cells were washed thrice with PBS and fixed with 4% paraformaldehyde at RT for 15 min. Cells were permeabilized in 0.25% Triton X-100 for 10 min at RT and blocked with PBS containing 2% bovine albumin sera plus 0.4% Triton X-100 at RT for 30 min. An in-house rabbit polyclonal to JEV nonstructural 1 (NS1) antibody at a dilution of 1:1,000 was added and incubated at room temperature for 1 h. Cells were washed thrice with PBS, as above, and then incubated with secondary antibody (goat anti-rabbit immunoglobin G conjugated with Alexa flour 568; Invitrogen) diluted 1:500 with PBS for 1 h at RT in the dark. After washing multiple times with PBS, cells were then washed thrice with PBS and mounted with a slow-fade gold antifade reagent with 4′, 6-diamidino-2-phenylindole (Invitrogen).

### Quantitative reverse transcription-polymerase chain reaction

N2a cells were plated in a 6-well plate at a density of 1.5 × 10^5^ cells per well. After 24 h, cells were infected with virus followed by transfection as previously described. After 48 hours of infection, cell total RNA was extracted by TRIzol Reagent (Invitrogen) following the manufacturer's instruction. Reverse transcription was performed using the cDNA synthesis kit (PrimeScript RT Kit; TAKARA). The polymerase chain reaction (PCR) was performed in Quanta RT PCR. Glyceraldehyde-3-phosphate dehydrogenase (GAPDH) mRNA expression levels were used for normalization. The primers used for JEV, GAPDH, interferon-stimulated genes (ISGs) such as 2′, 5′-oligoadenylate synthetase 1 (OAS1), IFITM1, and ISG15 interferon-stimulated gene 15, are shown in Table 2.

**Table 2.** List of Primers for Quantitative Real Time-Polymerase Chain Reaction

*Mouse*		*Sequence (5′-3′)*
*Oasl1*	Forward	CCAGGAAGAAGCCAAGCACCATC
	Reverse	AGGTTACTGAGCCCAAGGTCCATC
*Gapdh*	Forward	CCTGCCAAGTATGATGAC
	Reverse	GGAGTTGCTGTTGAAGTC
*Oasl2*	Forward	GGATGCCTGGGAGAGAATCG
	Reverse	TCGCCTGCTCTTCGAAACTG
*Isg15*	Forward	TGACGCAGACTGTAGACACG
	Reverse	TGGGGCTTTAGGCCATACTC
*Ifitm1*	Forward	CCTTCCTTATTCTCACTCTG
	Reverse	GTTGCAAGACATCTCACATC
JEV	Forward	AGAGCACCAAGGGAATGAAATAGT
	Reverse	AATAAGTTGTAGTTGGGCACTCTG

### Immunoblotting

N2a cells were plated in a 6-well plate at a density of 1.5 × 10^5^ cells per well. After 24 h, cells were infected with virus followed by transfection as previously described [6]. After 48 hours to transfection, cells were washed for 2 min thrice with PBS and lysed in cold lysis buffer (1% Triton X-100, 1 mM phenyl methylsulfonyl fluoride in PBS) for 1 h. The lysates were centrifuged at 12,000 *g* for 20 min. Total cell extracts were resolved by sodium dodecyl sulfate-polyacrylamide gel electrophoresis, transferred to nitrocellulose membranes, and then probed with an antibody (NS1, 1:5,000), followed by goat anti-rabbit IgG-HRP-conjugated antibody. GAPDH (1:5,000; GENTEX) was used as a loading control.

### Statistical analysis

All the experiments were performed thrice with each sample in triplicate and results were graphed, with error bars indicating the standard deviation. Statistical significance was determined using Student's *t*-test.

## Results

### Construction of amiRNA plasmids and cytotoxicity testing

**FIG. 2.**
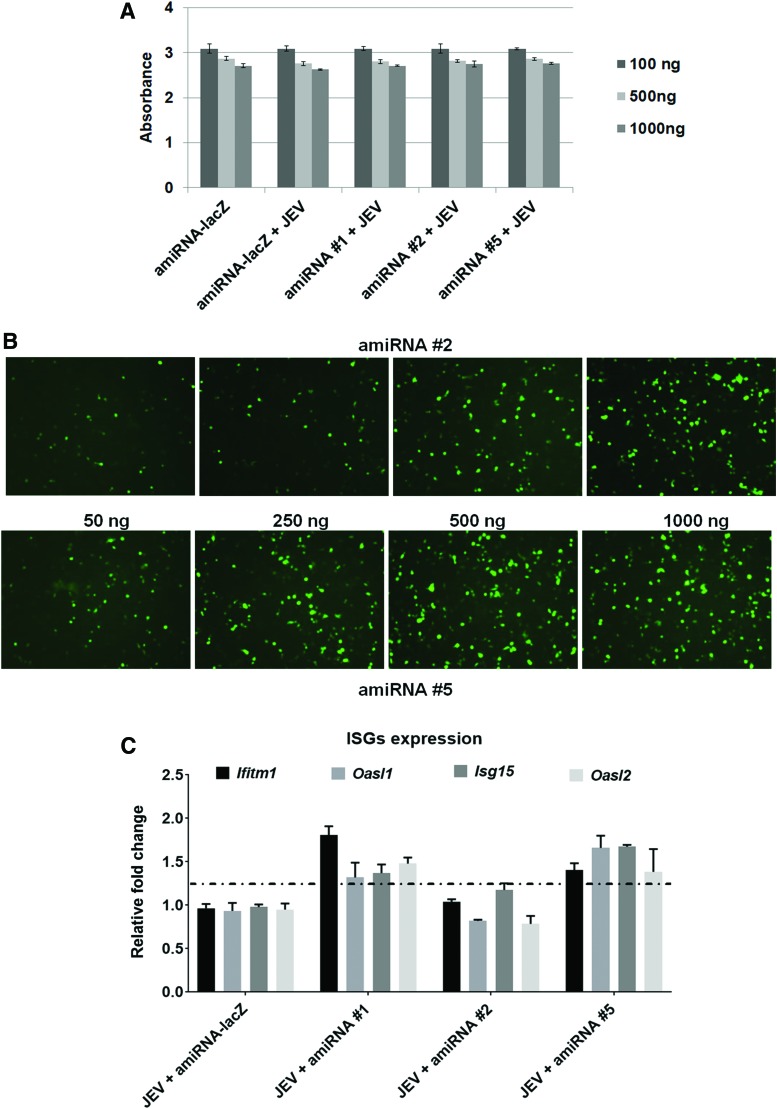
Transient transfection of amiRNAs and their effect on cell viability. **(A)** Cells seeded in a 96-well plate were infected with JEV at a MOI 5. Three hours postinfection, the cells were transfected with three different concentrations of amiRNAs (100, 500, and 1,000 ng) of single amiRNA per well. After 48 hpi, MTT reagent was added, and absorbance was measured at 570 nm. Results represent three independent experiments. **(B)** Cells were seeded in a 6-well plate and were transfected with four different concentrations of amiRNAs (50, 250, 500, and 1,000 ng) of single amiRNA per well. After 24 h, amiRNAs expression was monitored by checking eGFP expression under a fluorescence microscope. Representative images of amiRNA-treated HEK293T cells at 10 × magnification are shown. **(C)** RT-PCR analysis of four ISG (*Ifitm1*, *Oasl1*, *Oasl2*, and *Isg15*) mRNA expression. Gapdh gene expression was used to normalize the ISG expression. emGFP, emerald green fluorescent protein; hpi, hours postinfection; IFN, interferon; ISGs, IFN-stimulated genes; MOI, multiplicity of infection; qRT-PCR, quantitative real time polymerase chain reaction. Color images available online at www.liebertpub.com/nat

The amiRNA oligonucleotides were cloned into vector pcDNA6.2-GW/EmGFP-miR as recommended by the manufacturer's protocol. Briefly, to generate a construct that expresses two amiRNAs, we annealed top and bottom strands of amiRNA oligonucleotides and ligated them to another amiRNA backbone vector precut with *Bgl*II and *Xho*I. Using this approach, we prepared three JEV-specific amiRNA expression plasmids (amiRNA #1, amiRNA #2, and amiRNA #5) and an NC miRNA expression plasmid (amiRNA-lacZ). The positive recombinant plasmids were confirmed by nucleotide sequencing (data not shown). To determine the toxicity of amiRNAs in *in vitro* experiments, we performed the MTT assay (Promega) to evaluate the percentage of metabolically active cells after different transfecting concentrations of amiRNAs in N2a cells. 50–1,000 ng of plasmid vector harboring amiRNAs was transfected into N2a cells in each well of 96-well plates and incubated for 48 h. We did not observe significant toxic effect due to the presence of amiRNAs in cells (Fig. 2A). After transfection (∼24 h), fluorescence-positive cells were found, and green fluorescent protein (GFP) expression increased in a dose-dependent manner (Fig. 1B), suggesting that the transient transfection with EmGFP-amiRNA constructs was suitable as an indicator to test the transfection efficiency.

Exogenous miRNAs may be sensitive to interferon response and can exhibit substantial antiviral effect by inducing interferon-stimulated genes (ISGs). We checked four ISG (Ifitm1, *Oasl1*, *Isg15*, and Oasl2) expression in N2a cells after transfecting amiRNAs. We observed a variable level (but not significant) of ISG expression upon transfection of amiRNA #1 and amiRNA #5. However, no change in expression level of ISGs was evident in amiRNA #2-transfected cells.

### amiRNAs efficiently bind JEV 3′UTR and inhibit viral replication

**FIG. 3.**
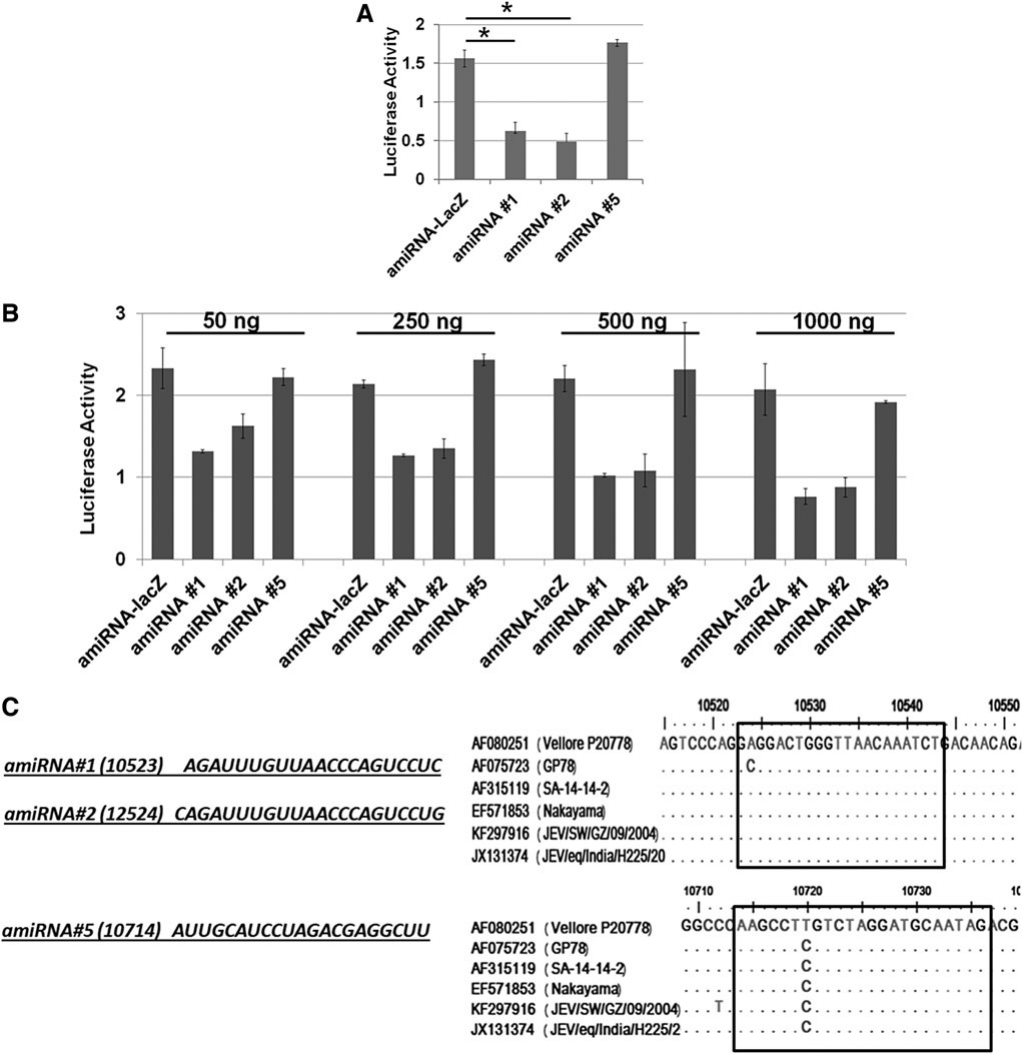
Binding of amiRNAs on 3*′*UTR of JEV Vellore strain (P20778) HEK293T cells were cotransfected with 100 ng JEV3*′*UTR luciferase construct and different concentration of amiRNA-expressing vector. After 48 h post-transfection, cells were lysed, and luciferase activity was measured. The data are shown as the firefly luciferase activity relative to the Renilla luciferase activity and are representative of three independent experiments. **(A)** Results represent the binding effect of 1,000 ng of amiRNA construct on JEV 3*′*UTR luciferase construct. The *asterisk* indicates statistical significance at 48 hpi (*P < 0.05). **(B)** Results represent the binding effect of different doses of amiRNA construct (50, 250, 500, and 1,000 ng) on JEV-3*′*UTR luciferase construct. **(C)** The nucleotide sequence of amiRNA #1, amiRNA #2, and amiRNA #5 (*left panel*) and their target site in JEV 3*′*UTR (*right panel*).

Like endogenous miRNAs, amiRNAs also exert their inhibitory function by binding the target sequence. To test the binding efficiency of our cloned amiRNAs with the target sequence, we cotransfected amiRNA clone (1,000 ng) and the JEV3′UTR-luciferase vector (100 ng) in HEK293 cells and measured the luciferase assay after 48 h of transfection. Transfection of amiRNA #1 and amiRNA #2 significantly reduced luciferase expressions, where amiRNA #5 did not have any binding effect on its target sequence and observed similar level luciferase activity as seen in control plasmid (Fig. 3A). We confirmed the results by transfecting amiRNA #1, amiRNA #2, and amiRNA #5 at different concentrations. As shown in Fig. 3B, transfection of 50 ng amiRNA clone reduces luciferase level up to 40% and luciferase level decreased in a dose-dependent manner. However, we did not observe any significant changes in the luciferase level with amiRNA #5. One possibility may be amiRNA #5 miRNA may not be able to express in our experimental condition. We checked GFP expression in amiRNA #5-transfected cells and found dose-dependent expression of GFP, suggesting that amiRNA expressed in the transfected cells (Fig. 2B). When we compared 3′UTR sequence of different JEV strain, we found nucleotide difference at 10720 positions (corresponds to the sixth position of the seed sequence of amiRNA #5) between P20778 Vellore strain and other strains (Fig. 3C). Since we are using Vellore strain for this study, the miRNA sequence may not be able to bind correctly to the 3′UTR region.

### Inhibitory effect of amiRNAs on JEV replication

**FIG. 4.**
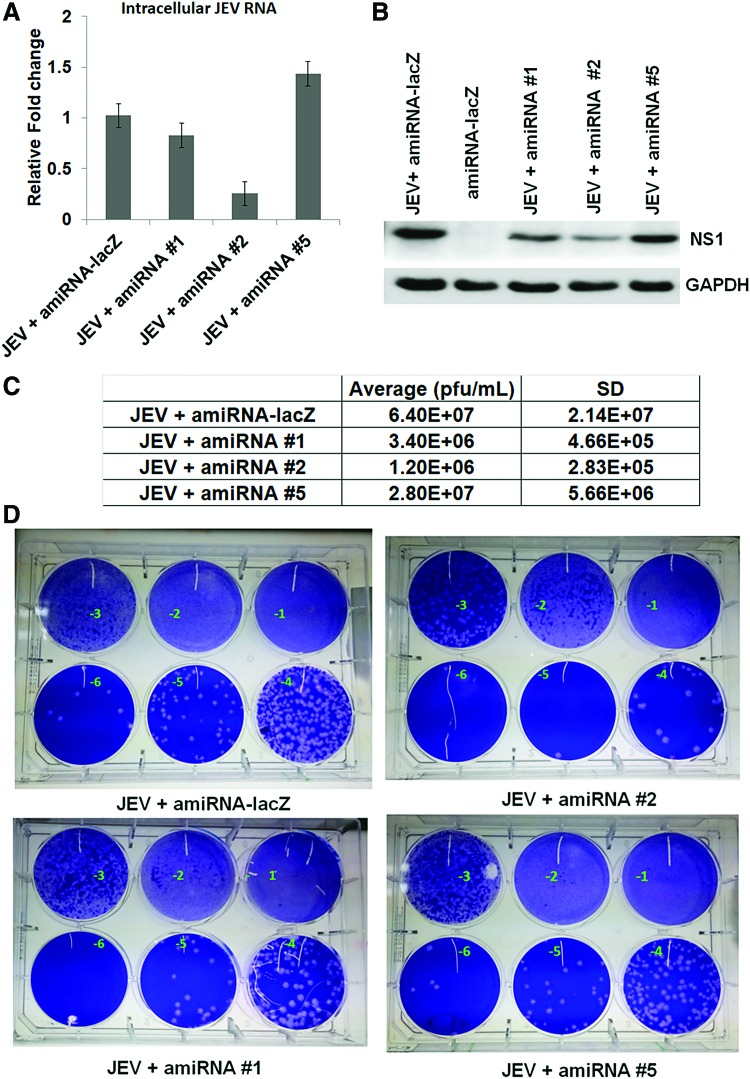
Effect of amiRNAs on JEV replication. Antiviral efficacy of each amiRNA was assessed in neuronal cells. **(A)** Determination of relative viral RNA by qRT-PCR. Cells were infected with cell culture-grown JEV (MOI = 5) and then treated with amiRNAs. After 48 hpi, cells were harvested, and RNA was isolated. The qRT-PCR was performed with specific primers for JEV region. Data represent the mean ± standard deviation of three independent experiments. **(B)** Western blots showing the amount of viral NS1 protein in neuronal cell lysate after treatment with different amiRNAs. Total cell lysate containing 30 μg proteins per sample was loaded. GAPDH served as an internal quantity and loading control. **(C)** Relative JEV titer in cell culture supernatant as measured by plaque assay at 48 hpi. **(D)** The 6-well plates are representing viral plaques after transfection of different amiRNAs. The number with a negative sign on each well represents 10 times dilution. GAPDH, glyceraldehyde-3-phosphate dehydrogenase; NS1, Nonstructural 1; qRT-PCR, quantitative real time polymerase chain reaction. Color images available online at www.liebertpub.com/nat

**FIG. 5.**
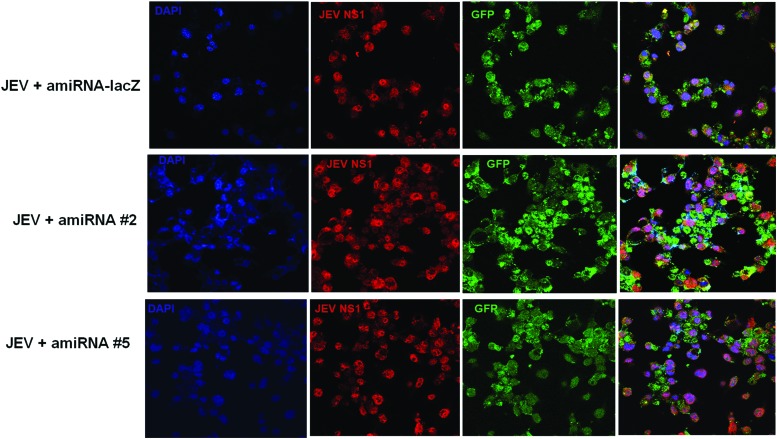
Reduced viral NS1 expression in neuronal cells by amiRNAs. Representative images of amiRNA-treated N2a cells showed staining of JEV NS1 protein. The *red fluorescence* indicates the virus load as assessed with anti-JEV NS1 mAb and a secondary antibody conjugated with Alexa-594, and *blue fluorescence* suggests the nuclear staining with DAPI. The *green fluorescence* represents amiRNA expression into the cells. Color images available online at www.liebertpub.com/nat

The efficacy of amiRNAs was evaluated by transfecting 1 μg of each plasmid into mouse neuroblastoma (N2a) cells after 3 hpi with JEV. We lysed the cells at 48 hpi for western blot (WB) analysis, and culture supernatant was used for plaque reduction analysis. Compared to control amiRNAs-lacZ and amiRNA #5, cells expressing amiRNA #2 exhibited a marked reduction in JEV RNA and NS1 protein expression as observed by quantitative real time-PCR (qRT-PCR) and WB at 48 hpi (Fig. 4A, B). Transfection of other amiRNAs showed the moderate effect on viral replication. Plaque assay was performed to quantify the infectious particles released into the culture supernatant. Compared to control, transfection of amiRNA #2 significantly reduced virus release in the culture supernatant (>95%). With other amiRNAs, a 20%–50% reduction was evident. JEV titer in amiRNA #1-, amiRNA #2-, and amiRNA #5-treated cells was 3.4 × 10^6^, 1.2 × 10^6^, and 2.8 × 10^7^ pfu/mL, respectively, at 48 hpi, compared to 6.4 × 10^7^ pfu/mL in virus amiRNA-lacZ, respectively (Fig. 4C). We also performed indirect immunofluorescence assay using amiRNA. Compared to control or amiRNA #5, cells transfected with amiRNA #2 showed a significant inhibitory effect. Our data revealed that cells with strong GFP exhibited less viral NS1 expression and cells expressing less GFP exhibited enhanced viral replication, as evidenced by NS1 staining in amiRNA #2-transfected cells (Fig. 5).

## Discussions

In this study, we examined the effect of vector-delivered amiRNA on JEV replication in neuronal cells. We have provided evidence that amiRNA-based RNAi could efficiently inhibit JEV replication in neuronal cells. This is the first report to successfully apply vector-delivered amiRNA targeted against the consensus sequence of JEV 3′UTR in inhibition of JEV replication. However, the efficacy of these amiRNAs remains to be tested *in vivo*.

Due to lack of proofreading activity of the viral polymerase, the RNA viruses are more prone to mutation in the open reading frame that sometimes hindered for developing an effective RNAi-based therapy against RNA viruses, particularly those that are neurotropic. Not only high rate mutation but also the presence of the blood-brain barrier raises significant concern in delivering the therapeutics in the brain. Several studies reported previously adopted a siRNA-based approach to inhibit JEV replication. However, synthetic dsRNA cannot pass the blood-brain barrier efficiently. An alternative method for the delivery of RNAi into the central nervous system (CNS) is required.

In our study, we have used the polymerase-II-promoter-driven plasmid vector that can produce amiRNA targeted against JEV 3′UTR [17]. This type of vector exhibited unique benefits in designing antiviral therapy as it provides comparatively less toxic RNAi molecules inside the cells. The effective amiRNAs can also be integrated into viral vectors, such as lentivirus, adenovirus, or adeno-associated virus, for delivery into the CNS [18]. Another way amiRNAs can also be delivered is by using naturally occurring small vesicles called exosomes. This exosome release from most of the cells may be engineered for delivering to specific tissues or organs [19,20].

For RNA viruses, mutations occur throughout the coding regions of its genome. In contrast, the 5′ and 3′ untranslated regions (5′ and 3′ UTR) display exceptional sequence conservation, as these regions play critical roles in translation and RNA replication initiation [21]. Taking advantage of this fact, we designed our amiRNA sequences from the conserved 3′UTR region. The amiRNA #2 seed sequences are highly conserved against all the virulent strains of different genotypes. We checked the RNAi activity of amiRNAs against Vellore strain (P20778). Due to the conservation nature of the seed sequence across the genotypes, we believe that amiRNA #2 will also be able to inhibit other viral strains of different genotypes efficiently. We did not find the significant inhibitory effect of amiRNA #5 even though the sequence cloned correctly and amiRNA #5 expressed in the cells. This may be because there is nucleotide mismatch between Vellore strain and the rest of the viral strain in the seed sequence region (Fig. 3C). This result further emphasizes the importance of complete seed match sequence, while designing the amiRNAs. Also, we concluded that the antiviral activity of amiRNA #2 was sequence specific as it did not induce an additional ISG response compared to control.

The amiRNA #1 also suppressed viral replication compared to amiRNA #5. Apart from the RNAi activity, the amiRNA #1 expression in the cells can induce ISG expression (especially IFITM1) as stated in Fig. 2C. IFITMs can restrict the replication of multiple viruses, including JEV [22,23]. Thus, the amiRNA #1-mediated effect as observed in our study may be the combined result of both sequences, specific as well as ISG-mediated response.

## Conclusions

Our data may support the use of a vector containing amiRNAs against different targets in 3′UTR of JEV. These findings offer a proof of concept for the development of novel amiRNA-based therapeutics against emerging JEV.
